# A review of experimental and computational attempts to remedy stability issues of perovskite solar cells

**DOI:** 10.1016/j.heliyon.2021.e06211

**Published:** 2021-02-12

**Authors:** Adam Kheralla, Naven Chetty

**Affiliations:** School of Physics and Chemistry, University of KwaZulu-Natal, Pietermaritzburg Campus, Private Bag X01, Scottsville 3209, South Africa

**Keywords:** Stability, Efficiency, Perovskite-Chalcogenide, Density function theory

## Abstract

Photovoltaic technology using perovskite solar cells has emerged as a potential solution in the photovoltaic makings for cost-effective manufacturing solutions deposition/coating solar cells. The hybrid perovskite-based materials possess a unique blend from low bulk snare concentrations, ambipolar, broad optical absorption properties, extended charge carrier diffusion, and charge transport/collection properties, making them favourable for solar cell applications. However, perovskite solar cells devices suffer from the effects of natural instability, leading to their rapid degradation while bared to water, oxygen, as well as ultraviolet rays, are irradiated and in case of high temperatures. It is essential to shield the perovskite film from damage, extend lifetime, and make it suitable for device fabrications. This paper focuses on various device strategies and computational attempts to address perovskite-based solar cells' environmental stability issues.

## 1 Introduction

**Figure 1:**
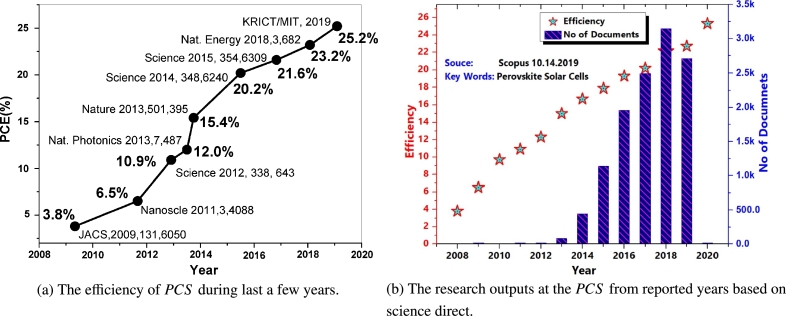
A gradual increase in performance of *PSC*[8], [9].

One of the critical problems confronting our community today is the need for eco-friendly and renewable energy sources to overcome the increasing energy demand regarding the swelling population and manufacturing. One of the promising technologies is solar cell technology, which is considered efficient for clean energy at low cost and minimal pollution [1]. The mineral perovskite was discovered and named after the Russian mineralogist Kon Lev Aleksevich von Perovski by Gustav Rose in 1839 after a sample was found in the Ural Mountains [2]. The compound named calcium titanium oxide (CaTiO_3_) describes a joint oxide group with a similar structure with the general formula $ABO_{3}$. Goldschmidt (1926) produced the first synthetic perovskites at the University of Oslo, leading to the term perovskite, which describes a class of compounds with the same general stoichiometry linkages as in CaTiO_3_ [3]. Recently, with appeared the fourth generation of photovoltaic technology, Perovskite Solar Cells (PSCs) have appeared, which exceeded expectations for Power Conversion Efficiency (*PCE*) within short term [4]. They attracted widespread attention from the solar cell research community due to their fantastic improvement in device efficiency with a significant increase from an initial value of 3.8% in 2009 [5], to 15% in 2013 [6], up to 23.3-25.2% recently [7]. As of 2020, a simple search on Science Direct on the titled perovskite-type solar cell production and characterization revealed a total of 1606 published papers. This indicates the high volume of the investigation carried out in the research field is shown in Fig. 1.

Despite the research efforts, a tiny portion of PSCs' gross research has reported power conversion efficiency greater than 25%. The reason is partly the instability of the perovskite medium and problems related to the devices remanufacturing [10]. Nevertheless, perovskite solar cell includes a structured compound with distinctive properties such as effective electroluminescence and photodetector applications [11]. It has a highly adjustable bandgap, a broad absorption spectrum, and simple fabrication [12]. Easy manufacturing techniques and low cost are advantages of perovskite devices compared to other silicon devices that make them promising for solid-state solar cells [13]. Perovskite has attracted widespread attention as a possible alternative to silicon photovoltaic devices currently dominate the photovoltaic market [14]. The instability of perovskite solar cells at the open-air environment is the main drawback of its large-scale realization and commercialization. Researchers have recently made considerable experimental and computational efforts to study the performance and control of the perovskite films' size, morphology, and crystallinity. This review inclusively discusses the evolution of the perovskite solar cells, details structures, and working. Addition to the experimental and computational design-fabrication, approaches of perovskite will discuss. Besides, we discuss some recent progress to resolve the stability challenges that affect perovskites device performance.

## 2 Architectures of perovskite solar cells

**Figure 2:**
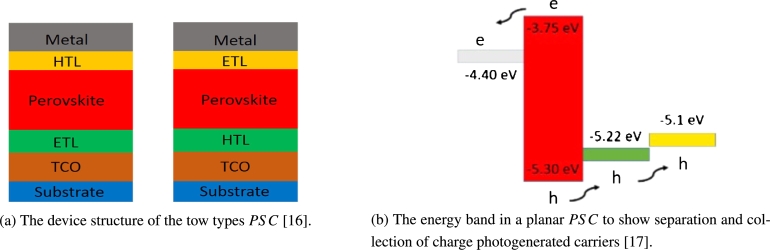
Architectures of perovskite solar cells [16], [17].

The comprehensive chemical structures of *PSC* are $ABX_{3}$, and $A_{2}BB^{\prime }X_{6}$, where *A* is an organic CH_3_NH_3_, NH_2_CHNH_2_, *B* is metal (Pb, Sn), *X* is halide, makes this material important for more applications [15]. PSCs include a perovskite photosensitive film confined between two electrodes. A surface buffer layer is usually used among the active and electrode layers to make easy charge processing. The device structure has two types of interface layers: Electron Transport Materials (*ETM*) and Hole Transport Materials (*HTM*). In fundamental, one of the electrodes should be a transparent conductive oxide, such as a nitride containing indium Fig. 2(a), to show the two PSCs' device structure. The structure of the device is widely used by depositing metal (top) electrodes of aluminium, silver or gold. There are two essential device architectures are used to prepare PSCs, mesoporous and planar *PSC* compositions. Therefore, the *PSC* charge transport channel is often discussed based on the kind of the device structure [15].

The perovskite layers have a mesoporous structure and are formed by a layer of porous semiconductor metal oxide such as titanium oxide TiO_2_, which forms an interlocking network between the two-phase interfaces. Therefore, light-induced electrons can be transported to the cathode through the TiO_2_ channel, and the pores are transported to the anode via the perovskite channel Fig. 2(b). At the planar structure, the interface hole layer and electron transport material are used to produce the cell. Excitons generated in the perovskite layer drifted into the electrode by an established electric potential or an externally imposed electric field.

## 3 Working principles of perovskite solar cell

Over the past a few years, several studies on charge transport kinetics in *PSC* have explained that light excitation in a perovskite medium will immediately generate electron-hole excitons in less than 2-picoseconds at electric field caused by the difference of function between anode and cathode and then splits it into delocalized charge carriers [18]. Besides, the success of perovskite solar absorbers mainly due to rising carrier mobility at the thin film medium, as well as charge spread the length of electrons and holes in the perovskite medium around 1 μm this is sufficient for the photogenerated charges reach into the interface layer and electrode without recombination [18]. Due to the complex nature of perovskite environment, researchers still do not know much about the process of generating and collecting charges in perovskite solar cells. So, the principle applied to characterize silicon solar cells is as yet used to describe PSCs' characteristics [19]. Typically, perovskite solar cells device can be described in four fundamental steps:

1. Photons absorption and excitons formation
2. Excitons diffusion and splitting
3. Charge transmission
4. Charge collection

**Figure 3:**
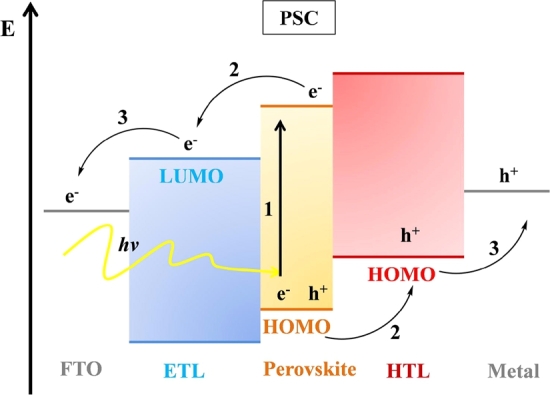
Work principles of Perovskite Solar Cells [20].

Once sunlight drop on the perovskite layer, it absorbs photons excitons. Owing to the difference in the excites' binding energies in the perovskite material, the exons can form electrons and holes when the excitons are separated. Exciton separation happens at the interface between the hole transport layer and the charge transport layer. Electrons are separated from the holes and injected into the electron transport layer, typically migrating to the Fluorine-doped Tin Oxide (FTO) anode, at the same moment, holes are mainlined into the holes transport layer and then transferred to the cathode, usually a metal as displayed in Fig. 3 [21]. The metal electrode collects electrons and holes, and the counter electrode is transferred to an external circuit to generate a current.

## 4 Perovskite based solar cells evolution

There has been the development of perovskites to achieved high the ideal efficiency for solar cells devices. Miyasaka et al. [22] reported the first perovskite solar cell in 2006 regarded by many as a benchmark towards achieving perovskite-based solar cell. They used CH_3_NH_3_PbBr_3_ just as the solar sensitive material and obtained a solar cell with an efficiency of 2.2% [23]. The first device from perovskite using TiO2 as sensitized solar cells was achieved by fluid electrolyte based on iodide [24]. Three years later, in 2009, Kojima et al. [25] reported the first organic lead halide synthesis CH_3_NH_3_PbBr_3_, CH_3_NH_3_PbI_3_ as sensitizers in the cell. The measurement of PSCs increased *PCE* approximately 3.81% into the CH_3_NH_3_PbBr_3_ as well as 3.13% into an CH_3_NH_3_PbI_3_-based cells, respectively, but with improved fabrication conditions. However, they found limitations produced by the nanocrystals' decomposition in the iodide fluid that led to quick device degradation. The device lasted only about 10 min [26]. In 2011, Park and colleagues MAPbI_3_ perovskite as quantum dots, resulting in enhanced productivity to 6.5% through TiO_2_ surface treatment. By mid-2012, Park et al. [27] tried to use solid organic molecules or polymers as an *HTM* and absorber (CH_3_NH_3_PbI_3_) into avoid an effect of electrolytes manufacture with a significantly increased the PCE of 9.7%. Demonstrates that a solid-state as *HTM* highly increased the device's stability compared with liquid electrolytes. Nevertheless, instability challenge remained the major limitation impeding manufacturing and photovoltaic marketing of PSCs [19]. In the same year, Snaith et al. [28] reported fabrications perovskite solar cells using Spiro-OMeTAD based *HTM* and Al_2_O_3_ as supports. The efficiency of the device was 10.9%. In their report, they showed that the use of mixed halides (CH_3_NH_3_PbI${}_{3x}$Cl_*x*_) can improve performance because of its higher charge carrying capacity. They also showed that perovskite has bipolar charge transport electrons and hole transport. In March 2013, Seok et al. [29] they found promising results from using the structures of nanoporous TiO_2_ infiltrated by mixed-halides *PSC* through optimization the halides in ${\text{CH}}_{3}{\text{NH}}_{3}\text{Pb}{\left({\text{I}}_{1-x}{\text{Br}}_{x}\right)}_{3}$ compound certified efficiency of 12.3%. Paves the way for reaching a PCE milestone that has been designed for many years. In 2013, PCE production of well-designed solar cells was 12.3% and 15%, increased to 19.3% in the first half of 2014 [30]. Seok et al. [31] reported an efficiency using CH_3_NH_3_PbI${}_{3x}$Cl_*x*_ and poly-triarylamine HTM of 16.2% and 17.9%, respectively. Subsequently, Saliba et al. [32] introduced a perovskite solar cell using a trication (Cs, MA, FA) mixture with 21.1% efficiency showed high stability and repeatability. Yang et al. [33] introduced a method to reduce defects in the perovskite layer using an intermolecular exchange process in 2017, which helps reduce the concentration of defects and achieve an efficiency of more than 22%. In 2018 Zhao et al. [34] all production reports of 4-terminal perovskite tandem solar cells with power conversion efficiency of over 23%. In 2019, Jiang et al. [35] produced a cell with an efficiency of 23.32% using organic halide HC(NH_2_)_2_CH_3_NH_3_ to prepared solar cells with surface defects. Sahil et al. [36] prepared fully textured monolithic perovskite-silicon tandem solar cell, and it achieved efficiency around 25.2%. Besides, to improving efficiency, a new design of device architecture has developed for low-cost and highly stable DSSC and *PSC*.

## 5 Band gap of perovskites

**Figure 4:**
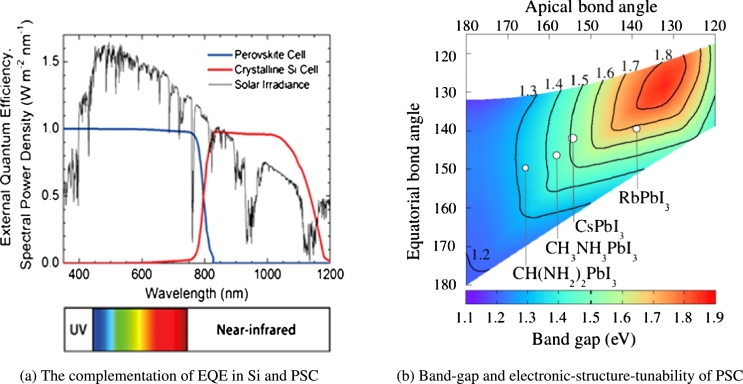
Sun irradiation and band gap of PSCs with permission from [38], [39].

**Figure 5:**
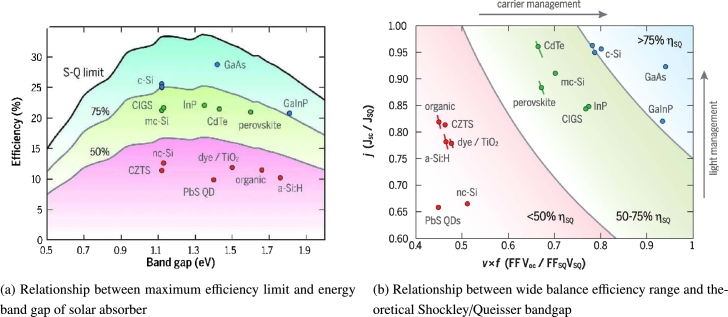
Solar cell efficiency limits and a fraction of Shockley-Queisser detailed-balance limit for V-J achieved by record cells [48].

The design of solar cell technology strongly depends on the knowledge of the energy from the sun. The sun releases a considerable amount of energy ≈174 PW, roughly 6000 times worldwide energy usage [37]. This energy is estimated to be 1366 W/m^2^ just outside the atmosphere Fig. 4(a) shows that upon integration, the range gives 1000 W/m^2^, using the energy equation of a photon Eq. (1) [40].

(1) $$E=\frac{hc}{\lambda }$$

where E is the photon energy, H is Plank constant, C is light constant and *λ* is the wavelength. That half the photons have a wavelength less than 1000 nm, which is between 1.24 to 1.5 eV [41]. Fig. 4(b) showed the bandgap and the relation between bond angles. The change in angular coordinates triggers a significant modulation of the bandgap from the top in the middle of infrared 1.1 eV to the starting of the visible spectrum 1.9 eV. This orientation is consistent with simple two-dimensional (2*D*) perovskite calculations that can handle electronics. The absorbed photons are photons with higher energy than the band gap of the material. Bandgap has an essential effect on the material's optical properties, in a systematic study Shockley and co. Workers found that most favourable bandgap should be near to the Shockley-Queisser limit 1.34 [42]. An important feature of the solar absorber is the bandgap. Determines the top power conversion efficiency theoretically, which is a natural feature that directly affects the actual performance of photovoltaic cells. In recent years, methylammonium lead iodide MAPbI_3_ has been widely used as a light absorber for PSCs with ideal bandgap. The long-term stability has currently been attributed to several studies, which will replace Methylammonium iodide (MA) MAPbI_3_. Although the best energy gap of a single-junction cell is between 1.1 and 1.4 eV, it has been reported that the bandgap of MAPbI_3_ is between 1.50 and 1.61 eV, further reducing PSC's solar power collection efficiency may be used MAPbI_3_ [43]. The substitution of MA ions and Formamidinium (FA) ions results in a slightly larger cubic structure, so the bandgap of MAPBI_3_ is 1.59 eV, and the bandgap of FAPbI_3_ is 1.45−1.52 eV, which is close to the best bandgap of a single-junction solar cell so that it can collect more light [44]. Between Cl, Br, and I, the absorbance changes significantly, with the increase of the halide ion size, and the energy gap, reduced, of single crystals, is US 2.97, US 2.24, and 1.53 eV is eV. Br and I parasite. Besides, compared with the absorption starter group, the smaller *PL* peak makes it should be used in solar cells advantageous [45]. Nowadays, the effect of cation exchange with MA^+^, Cs^+^ and Rb^+^ on the properties of FAPbI_3_ perovskite has been investigated through density function theory calculations. It was established that these additives might increase the energy gap of FAPbI_3_ [46]. R. Sa et al. [47] investigated by density function theory calculations the electronic and optical properties of Rb-doped MAPbI_3_, they found a band gap of Rb doped MAPbI_3_, between 1.53−1.49 eV for MAPbI_3_, Rb-doped systems, the band gap is 1.49 eV. Furthermore, it has been shown that Rb-doping will reduce the structural stability of MAPbI_3_, which can be attributed to the near band gap of it, which means if the bandgap <1 eV of a perovskites-type solar absorber is too small, the device may collect additional current from infrared emission, but the open-circuit voltage will be much smaller. Also, when the energy bandgap is more than 2 eV, a tiny amount from the sun's light can be harvested. Therefore, the materials with a bandgap of between 1.2−1.6 eV are excellent materials for the preparation of solar cells in the single-junction system's structural design, as shown in the Fig. 5. When the perovskite solar cells are composed of direct bandgap materials with low bulk trap densities, shown remarkable luminous properties, we can be adjusting bandgap in PSCs by molecular geometry.

### 5.1 Binding energy

**Figure 6:**
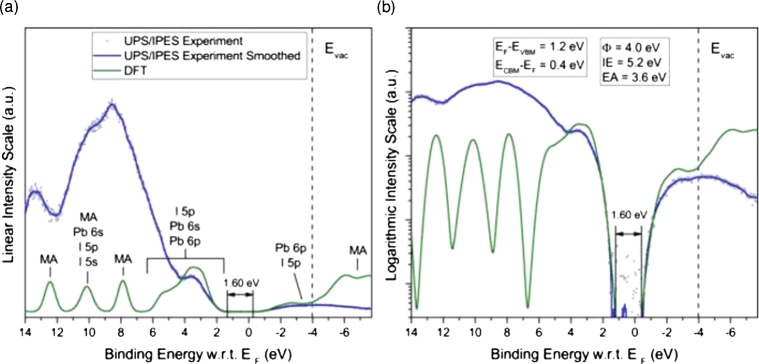
*UPS* with *IPES* spectrum of MAPbI_3_ in (a) linear, (b) semi-logarithmic graph. Compare this with the case density calculated by *DFT*[51].

It has already been mentioned that upon absorption of photons, *PSC* devices produce an exciton electrical bound pair. This exciton has to dissociate into electron and hole. Thus, the quantity of exciton binding energy has direct influences on *PCE* of the *PSC* device's performance. If the bending energy is weak, excitons are miss bound; then dissociation is favoured. While if band energy is elevation, excitons are tightly bound. Thus, recombination may be favoured [49]. The evaluation of the bending energy can be performed by carefully aligning the spectra obtained from the *PES* and IPES measurements with the simulated spectra were calculated from the *DFT* as displayed in Fig. 6 [50]. Endres et al. [51] have reported many significant improvements in the measurement of band onset a range by hybrid perovskite films, the band gap for MAPbI_3_ is determined should be 1.6±0.1 eV.

Another property that enhances charge dissociation is the degree of the modification in dipole moment from a ground state to the excited state [52]. The bigger the change, the higher the dissociation rate, and the inverse is true. The calculation is done by finding the dipole moment of molecules at ground state ($\mu_{g}$) and excited state ($\mu_{e}$). Then, using Eq. (2), the change in dipole moment can determine.

(2) $$▿\mu ={\left[{\left(\mu_{gx}-\mu_{ex}\right)}^{2}+{\left(\mu_{gy}-\mu_{ey}\right)}^{2}+{\left(\mu_{gz}-\mu_{ez}\right)}^{2}\right]}^{1/2}$$

## 6 Perovskite Chalcogenide

Perovskite-based on transition metal chalcogenides have emerged as a modern category of flexible semiconductors, with large chemical, as well as structural tenability that translates to a tenable bandgap visible to the infrared the electromagnetic spectrum [53]. In addition, this bandgap tenability provides a unique opportunity for realizing multi-state semiconductors with high carrier mobility. Recently, a series of transition metal perovskite chalcogenides $ABX_{3}$ (X = S, Se; A, B = metal) with low band gap have been theoretically predicted by *DFT* calculation and experimentally synthesized the reported performance is summarized based on the configurations displayed in Table 1. Some studies point out that most chalcogenides have the best band gap; therefore, band gap control is one of the keys to the perovskite device's stability. Meng et al. [61] utilized alloys with defect control in BaZrS_3_ compound investigated based on *DFT* method. The results of their calculations indicate that a small Zr substation $\text{Ti}(\text{Ba}{\text{Zr}}_{1-x}{\text{Ti}}_{x}{\text{S}}_{3},x=0.1)$ reduces the bandgap from 1.76 eV to 1.47 eV. Theoretically the *PCE* of $\text{Ba}{\text{Zr}}_{1-x}{\text{Ti}}_{x}{\text{S}}_{3}$ can exceed of the perovskite with the same thickness. They predicted that introducing compressive strain may be a plausible approach to stabilizing $\text{Ba}{\text{Zr}}_{1-x}{\text{Ti}}_{x}{\text{S}}_{3}$ perovskite film. The results show that some of the perovskite compounds based on the calculated Goldschmidt tolerance coefficient, band gap value, light absorption spectrum, effective mass, and phonon distribution [54] and expected to be used in photovoltaic applications. They also reported that materials mixing approaches can adjust the bandgap and light absorption of perovskite chalcogenides and can be used to design tandem photovoltaic devices [62]. The following section gives some details on methods to improve perovskite solar cells power conversion efficiencies through material modification.

**Table 1:** Summary of reported calculated bandgap by computational of perovskite solar cell materials (*ABX*_3_, X = S, Se).

Materials	Eg [eV] direct.	Eg [eV] indirect	Methods of study	References
SrSnSe_3_	1.56	1.56	*DFT*	[54]
SrSnS_3_	1.00	1.00	*DFT*	[54]
BaZrS_3_	2.25	2.25	*DFT*	[55]
BaZrSe_3_	1.44	1.01	*DFT*	[56]
CaZrSe_3_	1.52	1.52	*DFT*	[57]
LaYS_3_	1.79	1.79	*DFT*	[55]
CuTaS_3_	1.3	1.3	*DFT*	[58]
CsNbS_3_	1.47	1.47	*DFT*	[59]
CaSnS_3_	1.58	1.67	*DFT*	[60]

## 7 Design/fabrication approaches

Designing better perovskite electronic materials requires a comprehensive understanding of these materials electronic structure and the factors affecting it. By systematically studying a series of materials, valuable information can be generated regarding the materials properties. There are two distinct methods used to study the properties of materials, experimental and computational. Here we give a summary of both the experimental and computational approaches.

### 7.1 Experimental approach

Two main techniques are used to generate PSCs:

1. vacuum deposition
2. solution processing

**Figure 7:**
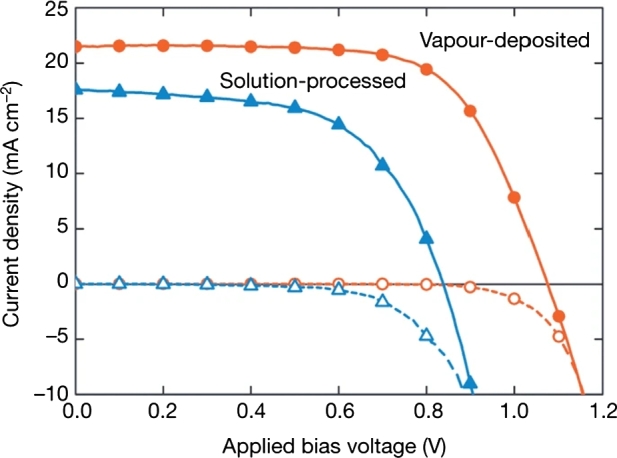
J-V characteristics produced by vapour deposition and solution deposition of the *PSCs*[6].

Although the first method has positive benefits, the second method is cost-effective and compatible with large-scale devices manufacturing. Researchers used different methods to design perovskite cells with different properties and reported power conversion efficiency Fig. 7. Dual first method (orange) and second method (blue) devices yields were 15.5% and 8.6%, respectively [63].

**Figure 8:**
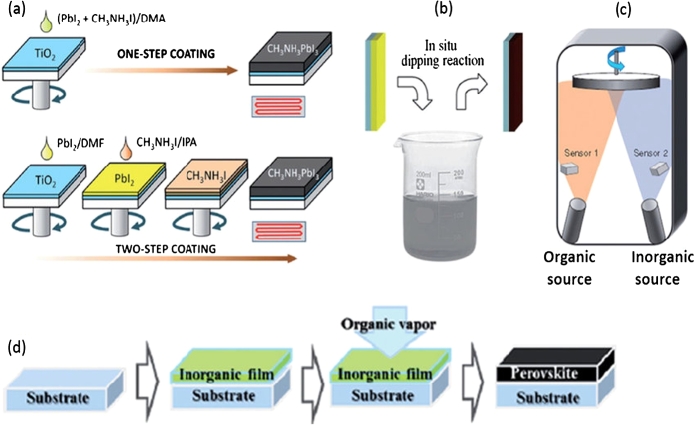
Different perovskite film preparation methods [16].

Typical methods commonly used in vacuum deposition are one-step precursor deposition, sequential vapour deposition, and dual-source vacuum deposition (TSVD). Among the solution deposition methods, so deposition method Fig. 8 shows (a) single-phase precursor deposition, (b) two-phase sequential deposition method, (c) DSVD and (d) VGP. Typically primary spin coating, secondary spin coating, vapour assisted solution deposition (VASD) and spraying [64].

Various experimental techniques such as X-ray diffraction [65], atomic force microscope [66], electron diffraction have been used, to better understand the structure of perovskite. Nevertheless, the determination of conformations in a suitable perovskite environment remains beyond the reach of available experimental techniques [67]. *Ab initio* calculations are one of the most promising computational approaches to obtain detailed structural information of such perovskite structure.

### 7.2 Computational approach

The computational approach is further divided into several disciplines, including molecular dynamics, chemical dynamics, molecular approaches, *ab initio*, and semi-empirical approaches. A widespread disadvantage of the above methods is that they depend on external parameters or experimental results. For example, as long as a constant force field of interaction parameters is provided between atoms, molecular dynamics simulations can predict molecules' structural evolution under temperature and kinetic properties [68]. Thus *MD* calculations strongly depend on *ab initio* calculation results, which assist into the improved force field and parameters necessary. Nowadays, *MD* successfully applied to the halide perovskites. Belugas et al. [69] employed the force field to study the vibration spectrum's temperature evolution and regenerated the essential characteristics of the vibration spectrum. The kinetic results of the *MD* simulation analysis show that the methylammonium lead iodide spectrum has a large temperature development, has the transformation of orthorhombic crystal to tetragonal crystal to cubic crystal, and strongly depends on the restriction and order of molecules. Taufique et al. [70] implemented a classic *MD* simulation to study the accumulation of PCBM, which forms a typical *ETL* on the surface of various perovskite crystals in the presence of solvents. There have been several reviews of the theoretical research into halide perovskites: reviews on optoelectronic properties [71], and Briefly reviews of the nature of chemical bonds [72], and electronic as well as ionic motions [73], reviews focusing within *MD* simulations [74]. *Ab initio* quantum mechanical calculation, which does not depend on external parameters, is of great help since it can predict the structure, energy levels, and optical transition strength of compounds before synthesis [75], [76].

#### 7.2.1 Density function theory

Since was introduced the Schrödinger equation [77], several excellent methods have been developed to solve electronic structure problems, including configuration interaction, Moller-Plesset perturbation theory, coupled-cluster expansion, and quantum Monte Carlo [78]. However, the matching Schrödinger equation among the coordinates in the multi-electron makes this method's calculation cost relatively high [79]. Density Functional Theory (*DFT*) began in 1926 with the appearing of the Thomas-Fermi theory [80], [81], an approximate method to discover atomic electronic structure using one electron at the ground state

density ρ(r) came as a remedy to the problem of solving coupled coordinates, thus reducing the computational costs. Hohenberg and Kohn-Sham [82] laid a solid foundation of the theory by mathematically precisely that of the real system, explain that in principle there is an exact method based on ρ(r), where the definition of ρ(r) is actually similar to the real system, currently *DFT* using the HK theorem [83]. The ground state energy can be written as in Eq. (3);

(3) $$E=T_{s}+U+V_{nuc}+E_{XC}[\rho ]$$

where $T_{s}$ is energy of Kohn-Sham, U is Hartree energy, $V_{nuc}$ the core and its attraction to and $E_{XC}[\rho ]$ is everything else that makes the above exactly. In the actual computations, the contribution of *XC* is approximate, and the result is only as good as the estimated value. The *XC* was first approximated using a theory called local density approximation (*LDA*), given by Eq. (4) developed in the 1970s but had many shortcomings, Generalized Gradient Approximation (*GGA*) represented by Eq. (5) were introduced, latter hybrids were introduced by Becke [84] leading to the well known and widely used exchange-correlation function called Becke's-three parameter Lee-Yang-Parr (B3LYP) [85].

(4) $$E_{XC}=\int \epsilon_{xc}(n)n(\overset{¯}{r})d^{3}r$$

(5) $$E_{XC}=\int (n,▿n)n(r)d^{3}r$$

**Figure 9:**
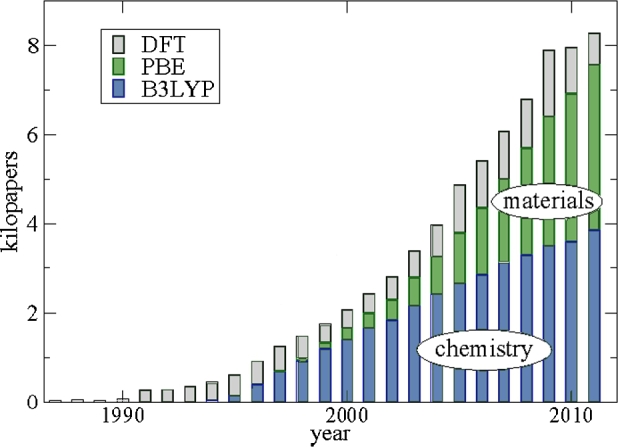
Number of *DFT* papers published based approximation function with citations [86].

Though *DFT* functionals were so well developed until the 1990s, they still could not treat Van der Walls interactions or provide accurate long-range dispenser forces [87]. The reason was that the binding energy curves decay exponentially as replacement of $-C_{6}/R^{6}$, where R is separation space, and $C_{6}$ is the Van Der Walls coefficient [88]. Different strategies for solving this problem have proposed: completely *ab-initio* approaches, re-parameterization of creating functions, and practical terms. Recently, the second method has attracted the interest of the people. Several attempts have reported that the term two-way affinity is used in molecular complexes and extended systems as $-f(R)C_{n}/R_{n}$ (n = 6, 7). The proposed correction varies in the damping function f(R) and the atomic-atomic distribution coefficient $C_{n}$. David Langreth and Bengal Lundquist [89] Developed an approximate non-local ground state density function LL, with the true decay [90]. Simultaneously, with this development, Grimme et al. [91] they developed the DFT-D methodology, which provides highly systematic and accurate experimental corrections for DFT results. The model suggests that it can successfully handle small molecule inserts, stacks and large complexes of biological systems such as DNA base pairs [92] and molecular crystals [93]. DFT use since its formalization has been steadily increasing, and better ways to treat chemical problems are emerging, as shown in Fig. 9 indicates a trend in the increase [86]. Up to this level, the success of DFT was only on ground-state electron structure calculations, without considerations of the excited-state [94]. The need to develop methods that treat excited states lead to the development of techniques like the main principle and ensembles [95]. Later a time-dependent DFT (TDDFT) was developed and has become a robust and viral method. The method is based on the fact that all observables in a quantum problem are functionals of the time-dependent density [96].

## 8 Instability of Perovskite Solar Cells

The primary challenge in realization perovskite solar cells today is the instability of the devices in a surrounding environment. Over the past few years, have made considerable effort to improve perovskite-type solar cell devices, stability by adopting various device architectures, compositions, and manufacturing technologies. Stability and efficiency are essential parameters for commercial applications of perovskite solar cells. Perovskite devices have achieved high efficiency so far. However, the stability of the devices is still one of the problems facing the research community. Perovskite compounds with manufacturing processes can contain traps into the degradation of perovskites [97]. The exposure of these layers to *UV* light [98], humidity [99], oxygen [100], and temperature [101] affect the stability of *PSC*. Various experimental and computational studies were carried out to determine possible solutions to improve stability. Solving the instability of perovskite materials is a key strategy to increase long-term stability. The geometric factors limit the formation of stable 3-dimensional the perovskite structures. Stability structure of perovskite can be defined by Goldsmidt's called tolerance coefficient, as shown in Eq. (6) [102].

(6) $$t=\frac{r_{A}+r_{0}}{\sqrt{2}(r_{B}+r_{0})}$$

**Figure 10:**
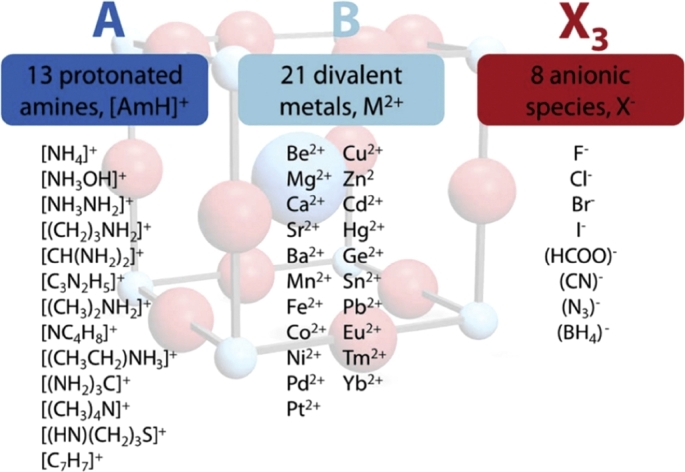
Calculated goldsmith's tolerance factor for some materials [112].

Here, $r_{A}$, $r_{B}$ and $r_{0}$ are organic cation A, inorganic cation B and halide anion X, respectively. The ideal cubic perovskites structure should be when t = 1 and the cubic structure can only be obtained when 0.89 less than t<t<1 [103]. A low tolerance factor means lowering symmetry, and the perovskite would shift to an orthorhombic or tetragonal structure, which would negatively affect the optoelectronic properties of perovskite [104]. The stability of perovskite materials most to be between a 0.8<t<1. So far the MAPbI_3_ is considered ideal stable perovskite which has a tolerance factor slightly higher than 0.9 [105], [106]. Computational approaches based on Goldschmidt tolerance factor were used to estimate the perovskite structure's geometric stability (three-dimensional) [107]. Filip and Giustino. [108] they applied a theoretical calculation based on density function theory was carried out on the entire periodic table. Korbel et al. [56] starting from over 32,000 possible 3D $ABX_{3}$ compounds as depicted in Fig. 10. Found suitable band gap values for 199 possible 3*D* $ABX_{3}$ thermodynamically stable perovskites with cubic structure and photovoltaic applications. The result of these studies is that lead has the best photoelectric properties in the context of the perovskite. Nevertheless, replacing toxic elements of Pb^+^ and Sn^2+^ while maintaining high performance is the main challenge for the research community [109]. Recently, chemical structure engineering or alloying has proven to be an effective strategy for adjusting the photoelectric and stable performance of perovskite-based solar cells, [110]. However, for lead-free perovskite systems, this method has not studied in depth. Further research may lead to the discovery of new lead-free perovskite [111].

### 8.1 Moisture instability

**Figure 11:**
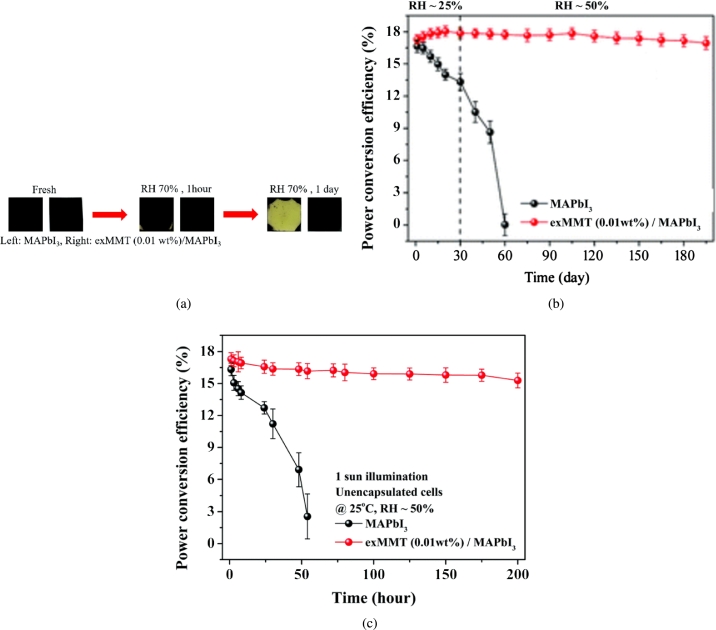
(a) Exfoliated Environmental stability of MAPbI_3_ films with and without passivation by exfoliated montmorillonite nanosheets; (b) power conversion efficiencies of MAPbI_3_ devices with and without passivation by exfoliated montmorillonite nanosheets film. (c) Relative humidity of the MAPbI_3_ at room temperature with constant sunlight [115].

Instability of perovskite by moisture arises, from the effect the hygroscopic nature on amine salt [113]. In addition, $\text{MA}\text{Pb}{\text{I}}_{3-x}{\text{Cl}}_{x}$ and MAPbI_3_ undergo humidity degradation process, in which the methylamine group is gone because of sublimation, PbI_2_ being further degraded, CH_3_, NH_2_ and *HI* [114]. Furthermore, recent experiments have shown that the formation of the MAPbI_3_ hydrate phase is a complete part of the degradation mechanism [99]. Huang et al. [115] used pure inorganic nanosheets as the photosensitive layer and MAPbI_3_ was added to the periscope precursor solution. As a result, nanosheets were slow down. The crystallization rate is reduced, forming larger particles and spheres on the perovskite's surface, effectively protecting the perovskite film from humidity, light, and thermal degradation [115]. New film stored at 70% relative ambient moisture did not change colour. At the same time, the control completely turned into yellow Fig. 11(a), Fig. 11(b), and (c), wherein the stability of the passivation device has greatly improved under humidity and light. A humidity, induced recombination mechanism is also proposed to control moisture synthesis in plane geometry. Although the efficiency has increased to 19.3%, it quickly drops below 5% of the actual performance when stored under environmental conditions [116]. The packaging technology developed for CIGS-based devices can effectively solve these devices humidity sensitivity [117].

**Figure 12:**
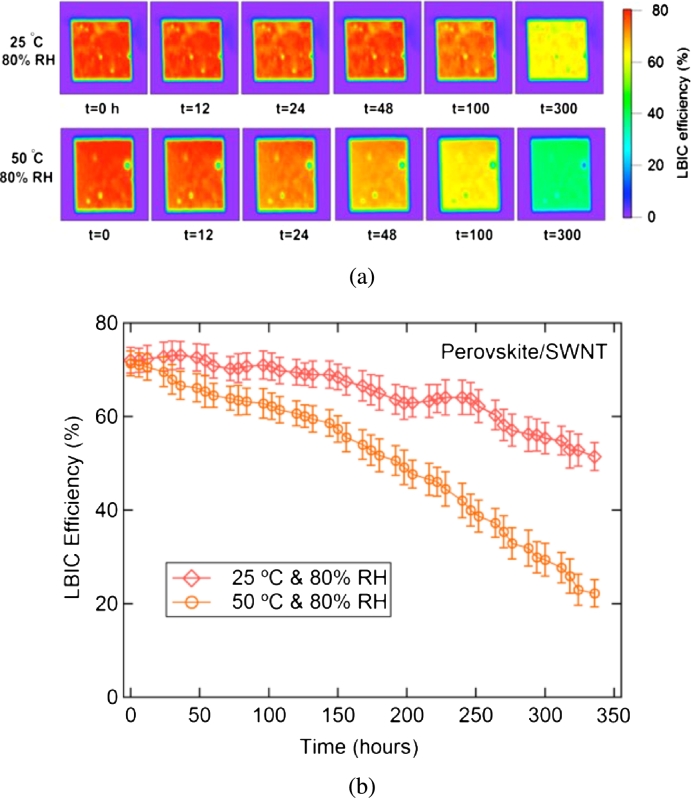
(a) LBIC mapping development of perovskite/SWCNT devices under high humidity conditions, (b) stability testing of perovskite/SWCNT devices under high humidity conditions [124].

Mosconi et al. [118] investigated by Ab Initio with Molecular Dynamics (AIMD) simulation for the interaction among the liquid water end surface of tetragonal MAPbI_3_ (001) with different terminations. Their results showed the interaction between Pb atoms and water molecules accelerate the release of I atoms. The surface of the MAI end is easy to dissolve. It was also found that, due to the stronger PbI bonds on these surfaces, the PbI^−2^ terminated surface is less sensitive to interfacial water, they also suggested that the incorporation of water molecules to the PbI_2_ exposed surface may be to the first step in the formation of an intermediate hydrated phase. Zhang et al. [119] applied *ab initio* molecular dynamics and *DFT* simulations of MAPbI_3_ perovskite, calculations results shown the light absorption attenuation caused by water is closely related to the formation of hydrated species, and the electronic excuses exposed to water. The adsorbed MAPbI_3_ nanoparticles tend to weaken the PbI bond in the excited state. Water molecules participate in electronic excitation, and binding mode is reduced into 20% due to light excitation, which is similar to that of perovskite. Denotes quickened of perovskite decomposition in the existence of sunlight and humidity. Long et al. [120] applied *ab initio* molecular dynamics from scratch. Found that the tetragonal MAPbI_3_ can destroy the perovskite surface with a small amount of adsorbed water on the surface of the MAI tip (001), bringing the photoelectrons closer to the surface. More importantly, by preventing overlapping, the electron holes are reduced by avoiding deep electron traps overlap and increasing the induced state's life span. Some of a theoretic studies efforts have focused on the effect of water molecules on the stability of different perovskite materials, such as MAGel_3_, MAPbI_3_ [121]. These studies establish that in MAPbI_3_, water readily diffuses to perovskite structure destructions, the formation of hydrated intermediates, and the humidity-related reduction in optical absorption observed in MAPbI_3_. Calculations based on *DFT* are used to study water degradation mechanisms in perovskite solar cells and propose strategies to improve water stability. Dong et al. [122] performed a systematic calculation on the identical molecular model of MAPbI_3_ and determined the stability of hydrogen bond interactions among the inorganic PbI_3_ and the organic CH_3_NH_3_ unit plays a vital role to determine the stability of MAPbI_3_. A computational investigation shows that due to the height polarity of water, the perovskite structure will, as a consequence, change in a humid environment. Depends on these theoretical insights, they deposited an ultra-thin Al_2_O_3_ film on the hole transport layer to improve the medium's stability without reducing efficiency. First-principles *DFT* calculations performed by Zhang and his colleagues on AIMD showed that the PbI_2_ end (001) surface of MAPbI_3_ is more stable than the MAI-terminated PbI_2_ defect surface in a humid environment [118], which is the same as calculated by Koocher et al. [123] They found that water adsorption on the surface of MAPbI_3_ (001) is greatly affected by the orientation of CH_3_NH_3_ (MA) cations close to surface. Water is positively adsorbed at all positions on the surface of PbI_2_ and MAI. Water can be actively adsorbed and supports the existence of the hydrated state observed in the experiment. This may be the first step in the corrosion process. The calculations (including the underlying release mode) indicate that higher water concentrations may promote degradation by increasing lattice deformation. Heben et al. [124] They Studied the degradation mechanism of perovskite devices against moisture. They added an SWCNT/PMMA composite as an encapsulation layer. They found that device resistance is increased against moisture with the encapsulation layer, and the average efficiency of the first-operation device is about 7%. Because of addition dielectric PMMA, the open-circuit voltage falls, and the series impedances increases significantly. These devices designed exhibit preferable current collecting uniformity and better breakdown resistance as displayed in Fig. 12. An increase in the SWCNT/PMMA package extends the device from 24 hours to 300 hours at high temperatures, while the device increases from 52 hours to 700 hours at low temperatures. By optimizing the production process and glass packaging applications, the service life of perovskite film can be extended SWCNT/PMMA electrodes with high heat resistance and moisture resistance and have considerable potential for the long-term stability of PSCs.

Thermal instability of PSCs is a significant problem for scientists community. When the device that subjects to a high temperature causing the device's degradation. Here, we discuss and summarise a few numerical and experimental studies of the effects of temperature on the PSCs, and strategies to improve the thermal stability of optoelectronic devices evolved. It is well known that temperature has a significant effect on crystal structure and perovskite phase. In a previous study on the effect of temperature, it was reported that the tetragonal phase became a cubic phase at 54 °C - 56 °C [125]. The performance (*η*) of the device depends to a large extent on the formula Eq. (7) reported in the literature [126].

(7) $$\eta_{c}=\eta_{Tref}[1-\beta_{ref}(T_{M}-T_{ref})]$$

**Figure 13:**
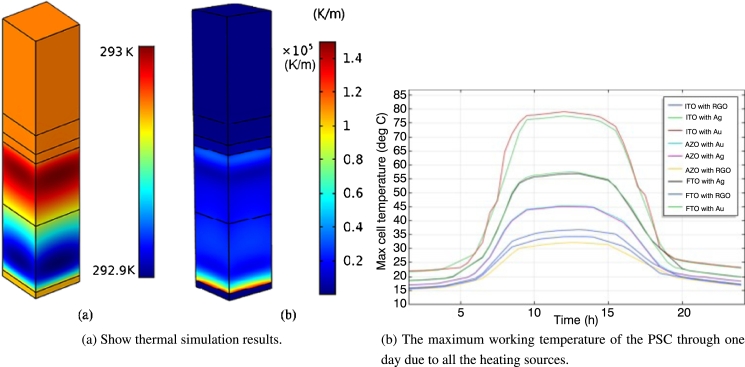
Simulation results of thermal instability of PSCs [127], [128].

*T* and *η* are the temperatures, respectively, and *PCE* and $\beta_{Ref}$ are the temperature coefficients of the module *PV*. It should be noted that these relationships reported for standard solar cell modules and that performance loss due to degradation of device components have not been considered. Makuleha et al. [127] applied numerical calculations to study the effect of thermal behaviour of a regular planar heterojunction perovskite solar cell made of Indium Tin Oxide (ITO) as the front plan and as a back contact (Au) on solar behaviour, solar radiation, heating and non-radiative recombination including joules. It was found that under the action of Joule heating, the maximum operating temperature of the battery reached 74.5 °C, non-radiative recombination was the smallest and largest, and solar radiation was the main source. The Fig. 13(b) shows the effect of all heat sources and the thermal behaviour of all combinations. The results suggest that the ITO/Au contact structure will have a higher operating temperature, reaching 79 °C. In comparison, due to higher emissions, the temperature of ITO/Ag will be slightly lower, around 77.7 °C. Subsequently, the FTO/Ag theme decreased by approximately 27.5%, and the FTO/Ag theme decreased by approximately the same percentage. High-temperature degradation resulted in the use of *AZO* as the front contact, Au and Ag as the back contact, and the temperature reached 45.3 °C [127]. Gorji et al. [128] applied the COMSOL calculations on the heat conduction between the perovskite solar cell and graphene. The results show that the bottom layer (RGO) temperature is lower than the other layers. It is used as a contact heat sink and a large conductor. The air medium is shown in Fig. 13(a). These results refer to the cell's improved thermal tendency due to the high thermal conductivity and thin thickness of the RGO layer.

**Figure 14:**
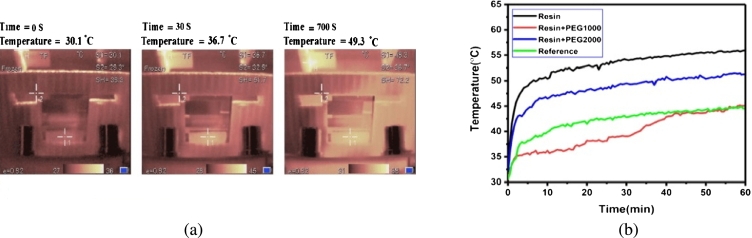
(a) Operating device temperature curve, (b) a temperature-time graph under the sun [129].

Fumani et al. [129] did design a polymer encapsulation layer both an adaptable humidity-blocked and temperature-controlled were used as cooling agent. Results have proved that the resin encapsulation process commonly applied in optoelectronic devices can be used as a barrier to heat release caused by light loss and electrical loss during devices operation. They also studied the effects of PSC temperature settings in a cooling system designed using four types of devices. These devices only use a reference cell attached to the sun's resin battery as a resin/PEG1000 and thermal control resin/PEG2000 system, and Fig. 14. In all the examples studied, the trend of temperature rise is shown in Fig. 14 (a), and (b). As seen, the maximum temperatures of the reference device, resin, resins/PEG1000 and resins/PEG2000 are 41.2, 49.3, 36.6 and 46.9 °C, respectively. Compared with uncalculated resins/PEG1000 and resins/PEG2000 encapsulated devices, pure resin encapsulated devices peaked. Electrical analysis results show that the device can be used for two years. After 450 days of storage, its reorganization and transport resistance has not changed much.

### 8.2 UV instability

Exposure to ultraviolet light is one of the main challenges that affect the stability of *PSC*, such as many another solar cell technologies, becomes cause degradation of *PSC* which attributed to using TiO_2_ as a photosensitizer in *PSC*, *UV* light can help the TiO_2_ could interact with $I^{-}$ and generate $I_{2}$, such as happen at DSSC. Therefore, it could destroy the perovskite crystal structure and enhance the ion reaction process of organic cations.

**Figure 15:**
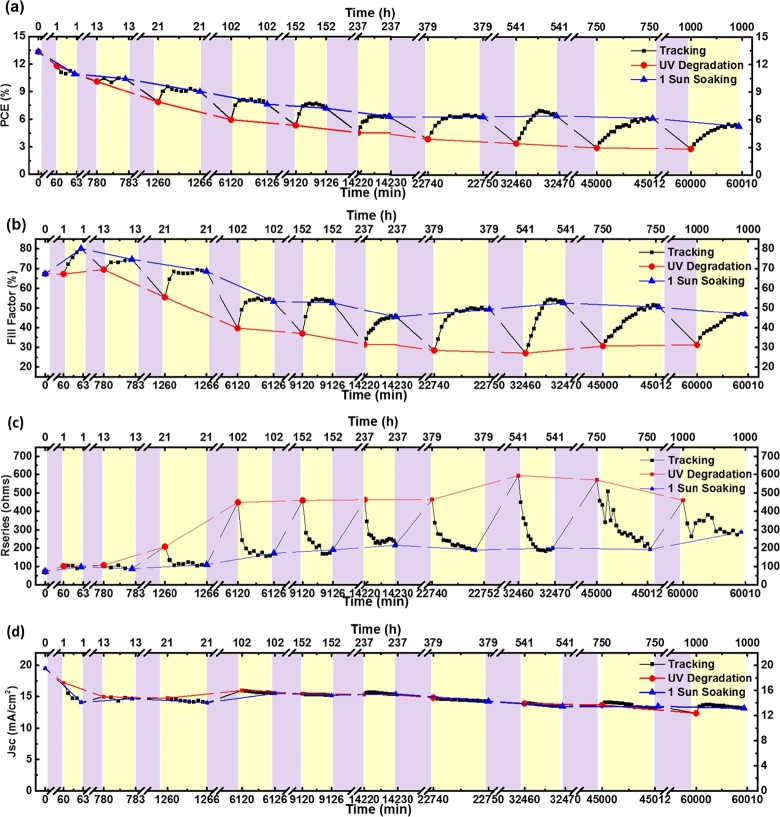
UV degradation/recovery cycle of perovskite device performance [130].

Leijtens et al. [131] was a study conducted to measure the efficiency drop curve of 5*h* for devices with packages and without packages and *UV* filters under one sun MM 1.5 G lighting conditions goes. The results show that the encapsulated device decreases faster than the unencapsulated device, indicating that degradation begins not only from the active layer but also from titanium dioxide TiO_2_, as depicted in Fig. 15. Display *UV* 1-sun solar bicycling light test results and possible mechanisms. Ito et al. [132] reported that the Sb_2_S_3_ layer in the interface among the mesoporous TiO_2_ and a perovskite layer increased stability due to the iodide pair's interruption at this interface. Unfortunately, UV filters can increase production costs due to additional material costs. It has also reported that the presence of oxygen prevents the ultraviolet activation degradation at the TiO_2_ interface, which eliminates the surface state and passivates the grave trapping sites at the interface, which is the titanium dioxide n-type semiconductor [131]. The use of aluminium silicate shells on titanium dioxide can increase stability [133]. With regards to (MSSC), the Al_2_O_3_ scaffold is a more stable alternative to TiO_2_ [134]. However, alternative charged layer and electrodes, interface engineering, and packaging technology must be sought into solving this problem. Various strategies have been reported to delay the UV-induced instability of *PSC* devices. Several groups have demonstrated the stability of about 1000 hours under a light with little or no degradation in performance [135].

## 9 Hysteresis effects

The hysteresis mechanism in perovskite devices is still unknown; several hypotheses were established. One of these hypotheses was ferroelectric polarization, widely discussed [136], and ion migration [137]. Hysteric behaviour could be dependent on the ferroelectric polarization. Thus, it is interesting to investigate hysteresis, depending on ferroelectric. Understanding the ferroelectric behaviour is essential to improve efficiency and stability because it affects light-stimulated electron-hole matching and separation [138]. Snaith et al. [139] suggested that the capacitive effect of the absorber, its ferroelectric properties and defect density are sources of hysteresis behaviour. If this effect is not taken into account, it will lead to an incorrect comparison between the efficiency value and the stabilized output [140]. The hysteresis observed in the current-voltage (J−V) curve is attributed to the ferroelectric fields under the applied electric field. The same effect can lead to ion migration or charge carrier trap [141].

**Figure 16:**
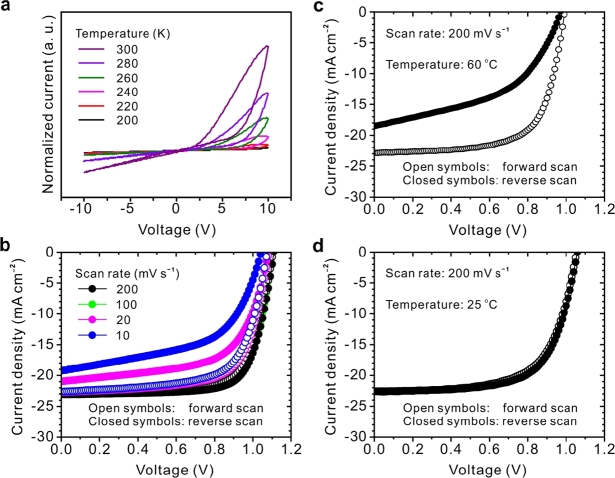
Diagram of ion migration in perovskites solar cells [142].

The presence of ion migration depends on measuring the temperature-dependent conductivity of the perovskite film in the lateral electrode structure and the current-voltage J−V curve. Ulzii et al. [142] used this design for ionic conduction; They found that ions do not contribute to conductivity at low temperatures, but when the temperature is high enough to provide the energy required to form mobile ions. Fig. 16 shows J−V curves, the results of these curves show that the hysteresis increased with rising temperature [143]. Recent studies have begun to back that charge trapping as well as ion migration may cause J−V hysteresis [144], [145]. Understanding the disruptive phenomena that cause degradation in the perovskite device is critical to improving efficiency and is an open space of research. Materials optimization and theoretic simulation through trial and error will ensure stable efficiency and contribute to technology maturity.

## 10 Conclusion and perspectives

In this article, we have reviewed the developments and fundamental structure the perovskite-based solar cells. We also explained in detail the several computational and experimental attempts to address and understanding certain vital factors such as the optical properties, band-gap energy, as well as the stability of the perovskites materials limiting development. The commonly used Pb element is highly toxic, and TiO_2_ suffers from UV-induced instability, contributing to the short lifetimes of *PSC*. Suggested alternatives, such as SnO_2_ and ZnO, have shown massive potential in the *PSC* community, with low-temperature fabrication. Moreover, replacing Pb with non-toxic ingredients will also prove beneficial in the future. We also introduce the perovskite-chalcogenide with narrow band gap, which provides more light absorption, one of the promising aspects of PSCs.

Currently, the theoretical approaches to understanding the microscopic physical mechanism of perovskite solar cells are considered a viable means. It will be easier to provide ideas for improvement and develop materials and structures that are simpler and more efficient if researchers can develop a theoretical device to verify the complex composition of the perovskite.

The instability issues of *PSC* are also still been debated, and more research is needed to full comprehension since this is a significant underlying problem of perovskite photovoltaic technology. Many strategies are being developed to improve the stability of PSCs against moisture, temperature, oxygen, and ultraviolet light. The stability of the perovskite device can effectively improve through advanced packaging technology against moisture and oxygen. Ultraviolet light and heat are unavoidable problems of perovskite devices during the operation. Given the rapid growth of *PSC* and a large number of publications, an investigation of newly designed strategies for improving the stability issues has taken a step forward in commercialization. Furthermore, more efforts should be made for the internal and basic mechanisms of the degradation operation. The significant operators influencing the degradation of perovskite in the environmental atmosphere should be elucidated to give more scientific orientation to improve stability.

## Declarations

### Author contribution statement

All authors listed have significantly contributed to the development and the writing of this article.

### Funding statement

This research did not receive any specific grant from funding agencies in the public, commercial, or not-for-profit sectors.

### Data availability statement

Data included in article/supplementary material/referenced in article.

### Declaration of interests statement

The authors declare no conflict of interest.

### Additional information

No additional information is available for this paper.
